# Viremia as a predictor of absence of serious bacterial infection in children with fever without source

**DOI:** 10.1007/s00431-022-04690-7

**Published:** 2022-11-18

**Authors:** Annick Galetto-Lacour, Samuel Cordey, Sebastien Papis, Chiara Mardegan, Fanny Luterbacher, Christophe Combescure, Laurence Lacroix, Alain Gervaix, Laurent Kaiser, Klara M. Posfay-Barbe, Arnaud G. L’Huillier

**Affiliations:** 1 Division of Pediatric Emergencies, Department of Women, Child and Adolescent Medicine, Geneva University Hospitals and Faculty of Medicine, Geneva, Switzerland; 2 Laboratory of Virology, Diagnostics Department, Geneva University Hospitals and Faculty of Medicine, Geneva, Switzerland; 3 Division of General Pediatrics, Department of Women, Child and Adolescent Medicine, Geneva University Hospitals and Faculty of Medicine, Geneva, Switzerland; 4 Division of Clinical Epidemiology, Department of Health and Community Medicine, Geneva University Hospitals and Faculty of Medicine, Geneva, Switzerland; 5 Division of Infectious Diseases, Department of Medicine, Geneva University Hospitals and Faculty of Medicine, Geneva, Switzerland; 6 Pediatric Infectious Diseases Unit, Department of Women, Child and Adolescent Medicine, Geneva University Hospitals and Faculty of Medicine, Geneva, Switzerland

**Keywords:** Biomarker, Viral systemic infection, Prognostic accuracy, C-reactive protein, Procalcitonin, Sensitivity, Predictor, Negative predictive value

## Abstract

Most children with fever without source (FWS) require diagnostic laboratory tests to exclude a serious bacterial infection (SBI), often followed by admission and empirical antibiotics. As febrile children with a viral infection are less likely to have a SBI, identifying patients with systemic viral infection could contribute to exclude SBI. We evaluated whether the presence of virus in the blood could be used as a biomarker to rule out SBI. Children < 3 years old with FWS were prospectively enrolled and had real-time (reverse-transcription) PCR performed on the blood for adenovirus, enterovirus, parechovirus, and HHV6. 20/135 patients had SBI, and in 47/135, at least one virus was detected in the blood. Viremia had a higher sensitivity and negative predictive value (90% and 96%) to rule out SBI compared to CRP (65% and 93%) and PCT (55% and 90%). The odds ratio (OR) for the presence of SBI among non-viremic patients was 5.8 (*p* = 0.0225), compared to 5.5 for CRP ≥ 40 mg/l (*p* = 0.0009) and 3.7 for PCT ≥ 0.5 ng/mL (0.0093). This remained significant after adjusting for CRP and PCT (OR 5.6 and 5.9, respectively; *p* = 0.03 for both). Area under the ROC curve for CRP and PCT were 0.754 and 0.779, respectively, but increased to 0.803 and 0.832, respectively, when combined with viremia.

*Conclusion*: The presence of viremia had a better performance than commonly used biomarkers to rule-out SBI and could potentially be used in conjunction with CRP and/or PCT in the evaluation of children with FWS. Larger studies should evaluate the role of point-of-care testing of viruses by (revere-transcription) PCR in the plasma in management algorithms of children with FWS.

**Table:** 

**What is Known:** *• Most children with FWS have a viral infection, but up to 15% have a SBI; most require laboratory tests, and many admission and empirical antibiotics.* *• Children with a viral infection are less likely to have a SBI.*	
**What is New:** *• Children with a systemic viral infection are less likely to have an SBI.* *• Viremia is a better predictor of absence of SBI than commonly used biomarkers and could potentially be used in conjunction with CRP and/or PCT in the evaluation of children with FWS.*

**Supplementary Information:**

The online version contains supplementary material available at 10.1007/s00431-022-04690-7.

## Introduction

Fever without source (FWS), defined as temperature ≥ 38.0 °C with no cause identified after a thorough medical history and clinical examination [1], is one of the most frequent reasons for pediatric emergency department (PED) visits [2]. Because 9–18% of the children presenting with FWS have a serious bacterial infection (SBI) [3–6], many children require diagnostic laboratory tests to identify the few patients with SBI, followed by admission and empirical administration of broad-spectrum antibiotics. This is especially challenging in younger patients who often have a non-specific clinical presentation and are at increased risk of SBI [1, 3, 7, 8].

Even though most children with FWS have a self-limited viral infection, laboratory investigations performed in this context are meant to rule in or rule out SBI. For example, biomarkers, such as procalcitonin (PCT) and C-reactive protein (CRP), have been shown to be good predictors of SBI [9–11]. Similarly, the Lab-score, designed to assist clinicians in decision-making, permits an estimation of the risk of SBI [9–13]. Previous data show that febrile children with a documented viral infection are less likely to have a concomitant SBI [4, 14–21]. Identifying patients with a systemic viral infection could therefore contribute to exclude SBI. In this study, we aimed at evaluating whether the presence of viremia could predict the absence of SBI in children with FWS.

## Patients and methods

### Study design

This is a substudy of a prospective, single-center, epidemiological diagnostic study [4]. Patients 0–3 years old with a clinical diagnosis of FWS were enrolled in the PED of a tertiary center (Geneva University Hospitals). Exclusion criteria were comorbidities predisposing to infections such as cancer, primary or secondary immunodeficiency, and iatrogenic immunosuppression). Besides the standard institutional FWS protocol (Supplementary Methods) which was based on published evidence [9, 22–25], enrolled children had blood systematically sampled for the detection in plasma of human enterovirus (HEV), human parechovirus (HPeV), adenovirus (AdV), and human herpesvirus type 6 (HHV6) by real-time (reverse-transcription)-polymerase chain reaction [4]. Patient information was recorded on individual anonymized case-report forms designed for this study, as previously described [4].

### Groups

Study patients were divided into two groups based on the diagnosis of SBI. SBIs consisted of documented bacteremia requiring antibiotic treatment (blood cultures interpreted as contaminant were not considered as SBI), bacterial meningitis, osteomyelitis, pneumonia, or urinary tract infection (UTI). UTIs were defined as a positive urinalysis and ≥ 10e5 CFU/ml of a single uropathogenic organism in appropriately collected specimens, as per the American Academy of Pediatrics [26]. More details about UTI definitions are provided in the supplementary methods of the main manuscript [4]. Patients were considered viremic if found positive for at least one of the following viruses in the plasma: HEV, HPeV, AdV, and HHV6.

### Ethics

This study was approved by Geneva’s Ethics Committee (CCER #15-082) and registered under Clinicaltrials.gov (NCT03224026​). No investigation was performed before signature of the informed consent.

### Statistics

Continuous variables were compared using the Mann–Whitney test. Dichotomous variables were compared using Chi-squared or Fisher exact test. Proportions are reported with 95% confidence intervals (Clopper-Pearson exact method). Univariate and bivariate logistic regression was used to evaluate biomarkers performance individually and in combination. The combination CRP + viremia was obtained with a multivariable logistic regression model: CRP and viremia were linearly combined using the regression coefficients. The same was true for the combination PCT + viremia. Odds ratios assessed with logistic regression models are reported with 95% confidence intervals. Receiver operating characteristic (ROC) curves with area under the curve (AUC) were also used to evaluate biomarkers performance (non-parametric approach). *p* values < 0.05 were considered significant. Statistics were calculated using SPSS software, version 23.0 (IBM Corp., Armonk, NY), and all statistical tests were two-sided.

## Results

### Demographics

One hundred and thirty-five patients enrolled between November 1, 2015, and December 31, 2017, had blood tested for HEV, HPeV, AdV, and HHV6. Among those, 20 were diagnosed with SBI. Most SBIs consisted of UTIs (*n* = 18), followed by *H. influenzae* (type f) bacteremia with concomitant meningitis (*n* = 1) and *P. aeruginosa* meningitis (no known comorbidities). The demographics of study patients are detailed in Table S1, and discharge diagnosis of patients without SBI is described in Table S2.

### Viral systemic infection

Among the study cohort, 47 patients had a least one virus detected by real-time (reverse-transcription [RT]) polymerase chain reaction (PCR) in the plasma. Namely, HEV was detected in 19 patients, HHV-6 in 15, HPeV in 8, and AdV in 7 (co-infection AdV/HEV and AdV/HPeV in one patient, respectively). Within the dataset, two patients had a concomitant SBIs and viral systemic infection (UTI + HEV; UTI + HHV6).

### Performance of biomarkers

There were significantly less viremic patients in patients with SBI than in those without SBI (*p* = 0.011) (Table S1). Similarly, there were significantly more patients with a CRP ≥ 40 mg/l or a PCT ≥ 0.5 ng/ml among patients with SBI (*p* < 0.001 and 0.007, respectively) (Table S1).

Viremia had a higher sensitivity and negative predictive value (90% and 96%, respectively) to rule out SBI when compared to CRP < 40 mg/l (65% and 93%, respectively) and PCT < 0.5 ng/ml (55% and 90%, respectively) (Table 1). Taking each virus individually, the absence of viremia had sensitivities and negative predictive values ranging between 95–100% and 93–100%, respectively (Table 1). Sensitivity and negative predictive values were further improved compared to CRP and PCT in the subgroup of children < 3 months, when compared to the whole dataset or the subgroup ≥ 3 months (Table 1).

**Table 1:** Performance of CRP, PCT and viremia to discriminate patients with or without serious bacterial infection

	**Sensitivity** **%** (n/N) *95% CI*	**Specificity** **%** (n/N) *95% CI*	**Positive** **predictive value** **%** (n/N) *95% CI*	**Negative** **predictive value** **%** (n/N) *95% CI*	**Positive** **likelihood ratio** *95% CI*	**Negative** **likelihood ratio** *95% CI*
**Full dataset**						
** CRP ≥ 40 mg/l**	**65.0** (13/20) *40.8 − 84.6*	**74.8** (86/115) *65.8 − 82.4*	**31.0** (13/42) *17.6 − 47.1*	**92.5** (86/93) *85.1 − 96.9*	**2.58** *1.64 − 4.04*	**0.47** *0.26 − 0.86*
** PCT ≥ 0.5 ng/ml**	**55.0** (11/20) *31.5 − 76.9*	**75.0** (84/112) *65.9 − 82.7*	**28.2** (11/39) *15.0 − 44.9*	**90.3** (84/93) *82.4 − 95.5*	**2.20** *1.32 − 3.66*	**0.60** *0.37 − 0.99*
** No viremia**	**90.0** (18/20) *68.3 − 98.8*	**39.1** (45/115) *30.2 − 48.7*	**20.5** (18/88) *12.6 − 30.4*	**95.7** (45/47) *85.5 − 99.5*	**1.48** *1.20 − 1.82*	**0.26** *0.07 − 0.97*
** No HEV viremia**	**95.0** (19/20) 75.1* − 99.9*	**15.7** (18/115) *9.5 − 23.6*	**16.4** (19/116) 10.2* − 24.4*	**94.7** (18/19) *74.0 − 99.9*	**1.13** *0.99 − 1.28*	**0.32** *0.04 − 2.26*
** No HHV-6 viremia**	**95.0** (19/20) 75.1* − 99.9*	**12.2** (14/115) *6.8 − 19.6*	**15.8** (19/120) 9.8 − 23.6	**93.3** (14/15) 68.1* − 99.8*	**1.08** *0.96 − 1.22*	**0.41** *0.06 − 2.95*
** No HPeV viremia**	**100.0** (20/20) 83.2* − 100.0*	**7.0** (8/115) 3.1* − 13.2*	**15.7** (20/127) *9.9 − 23.3*	**100.0** (8/8) 63.1* − 100.0*	**1.08** *1.02 − 1.13*	**-**
** No AdV viremia**	**100.0** (20/20) 83.2* − 100.0*	**6.1** (7/115) 2.5* − 12.1*	**15.6** (20/128) *9.8 − 23.1*	**100.0** (7/7) 59.0* − 100.0*	**1.07** *1.02 − 1.12*	**-**
**Age < 3 months**						
** CRP ≥ 40 mg/l**	**53.8 **(7/13) *25.1 − 80.8*	**90.9** (60/66) *81.3 − 96.6*	**53.8** (7/13) *25.1 − 80.8*	**90.9** (60/66) *81.3 − 96.6*	**5.92** *2.37 − 14.77*	**0.51** *0.28 − 0.92*
** PCT ≥ 0.5 ng/ml**	**46.2 **(6/13) *19.2 − 74.9*	**86.4 **(57/66) *75.7 − 93.6*	**40.0** (6/15) *16.3 − 67.7*	**89.1** (57/64) *78.8 − 95.5*	**3.38** *1.45 − 7.88*	**0.62** *0.37 − 1.04*
** No viremia**	**92.3** (12/13) *64.0 − 99.8*	**34.8** (23/66) *23.5 − 47.6*	**21.8** (12/55) *11.8 − 35.0*	**95.8 **(23/24) *78.9 − 99.9*	**1.42** *1.12 − 1.79*	**0.22** *0.03 − 1.49*
**Age ≥ 3 months**						
** CRP ≥ 40 mg/l**	**85.7** (6/7) *42.1 − 99.6*	**53.1** (26/49) *38.3 − 67.5*	**20.7** (6/29) *8.0 − 39.7*	**96.3** (26/27) *81.0 − 99.9*	**1.83** *1.19 − 2.79*	**0.27** *0.04 − 1.68*
** PCT ≥ 0.5 ng/ml**	**71.4** (5/7) *29.0 − 96.3*	**58.7** (27/46) *43.2 − 73.0*	**20.8 **(5/24) *7.1 − 42.2*	**93.1** (27/29) *77.2 − 99.2*	**1.73** *0.97 − 3.09*	**0.49** *0.15 − 1.61*
** No viremia**	**85.7** (6/7) *42.1 − 99.6*	**44.9** (22/49) *30.7 − 59.8*	**18.2** (6/33) *7.0 − 35.5*	**95.7** (22/23) *78.1 − 99.9*	**1.56** *1.05 − 2.31*	**0.32** *0.05 − 2.01*

*CRP* C-reactive protein, *PCT* procalcitonin, *CI* confidence interval, *HEV* human enterovirus, *HHV-6* human herpesvirus type 6, *HPeV* human parechovirus, *AdV* adenovirus

Using univariate logistic regression models, the odds ratio (OR) for having SBI among non-viremic patients was 5.79 (95% CI 1.57–37.50; *p* = 0.0225), compared to 5.51 for CRP ≥ 40 mg/l (95% CI 2.06–15.93; *p* = 0.0009) and 3.67 for PCT ≥ 0.5 ng/ml (95% CI 1.38–10.00; *p* = 0.0093) (Table S3). Similarly, the OR for SBI increased with CRP and PCT levels (*p* = 0.0002 and < 0.0001, respectively) (Table S3). Using bivariate logistic regression models, the OR for having SBI among non-viremic patients was 5.59 (95% CI 1.39–38.42) and 5.94 (95% CI 1.41–43.45) times higher than among viremic patients after adjusting for CRP and PCT, respectively (*p* = 0.03 for both) (Table S3). After adjusting for viremia, the OR for having SBI increased with CRP and PCT levels (*p* = 0.0004 and < 0.0001, respectively) (Table S3).

**Fig. 1:**
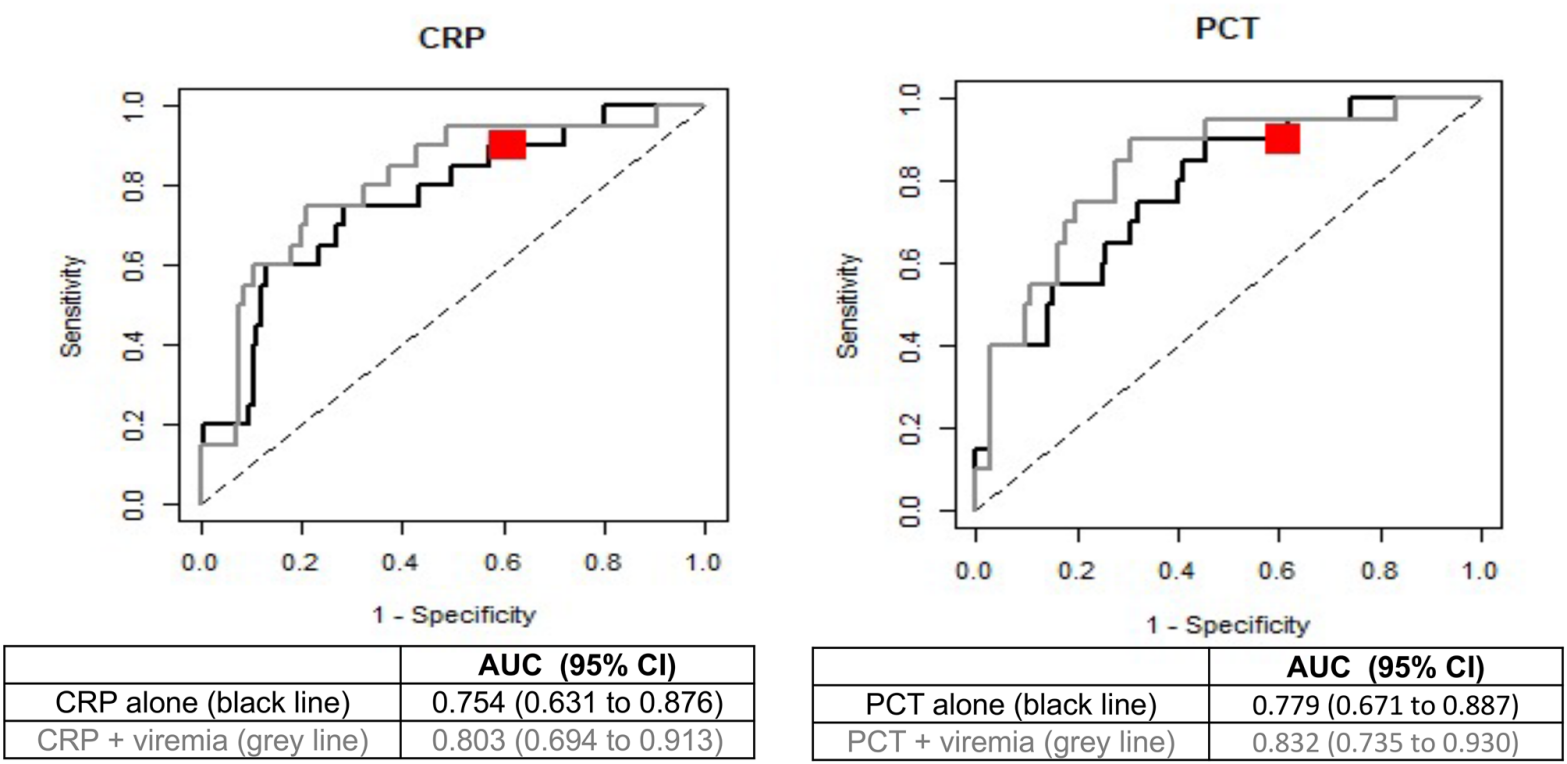
ROC curves for the performance of CRP and PCT alone or in combination with viremia in discriminating patients with or without SBI. ROC, receiver operating characteristic; CRP, C-reactive protein; PCT, procalcitonin; SBI, serious bacterial infection; AUC, area under the curve; CI, confidence interval. Black ROC curves represent CRP and PCT alone, whereas grey lines represent CRP and PCT in combination with viremia. The red Dot corresponds to the performance of viremia alone

The AUC for CRP and PCT were 0.754 and 0.779, respectively (Fig. 1). When viremia was combined to CRP and PCT, the AUC increased to 0.803 and 0.832, respectively (Fig. 1). Subgroup analyses in children < 3 months and ≥ 3 months confirmed a superior AUC if viremia was combined to CRP or PCT compared to CRP or PCT alone (Table S4).

## Discussion

This study evaluated the prognostic accuracy of the absence of viremia as a predictor of SBI in children with FWS. The main finding was that the sensitivity and negative predictive value of viremia compared to commonly used biomarkers such as CRP and PCT allowed to better rule out an SBI. This remained true whether viruses were analyzed together or individually. The superiority of viremia when compared to CRP or PCT was particularly evident in children < 3 months, which is important because most of them will require blood testing given their higher likelihood of SBI. Similarly, the likelihood of SBI was approximately six times lower among viremic patients, even after adjusting for CRP and PCT. Using ROC curves, CRP and PCT displayed better prognostic accuracies when combined to viremia, confirming the independent prognostic value of the absence of viremia and its potential interest when used in conjunction with CRP and/or PCT. These findings are in line with previous evidence showing that febrile children with a documented viral infection are less likely to have a concomitant SBI [4, 14–21]. The risk reduction in bacterial infection among virus-infected children differs between viruses and the anatomical specimen where the virus is identified. Indeed, asymptomatic carriage of viruses is very common in the respiratory [27, 28] and digestive [29, 30] tracts, especially in younger children and consequently strongly limits the sensitivity to rule out SBI. For example, the detection of human rhinoviruses in respiratory specimens does not reduce the likelihood of bacterial infection to the same extent than other respiratory viruses [21, 31], most likely because of the frequency of nasopharyngeal carriage reaching up to 33% for human rhinovirus among children < 36 months [28]. For those reasons, viruses detected in the blood are more likely to be clinically relevant than those detected in the respiratory or gastrointestinal tract, and hence the sensitivity of viral studies in blood to rule out SBI will be higher than in the respiratory or gastrointestinal tracts. Given the relatively low proportion of SBI among children with FWS, optimized sensitivity is of interest since it better allows to rule out SBI. Therefore, it was outside the scope of the current study to compare viral shedding patterns between blood and other anatomical specimens.

With the SARS-CoV-2 pandemic, there has been a dramatic development of point-of-care testing (POCT) RT-PCR testing, with turnaround times as fast as 20 min [32, 33]. In addition to currently performed diagnostic tests, the development of POCT (RT)-PCR for the diagnosis of systemic viral infections could be an additional to help clinicians in the evaluation and management of children with FWS. Furthermore, it could also potentially help to reduce hospital admissions and empirical prescription of broad-spectrum antibiotics, which could indirectly reduce healthcare-associated costs and counterbalance the costs of (RT)-PCR testing.

This study has some limitations. First, only four viruses were tested. However, those viruses were selected because of (1) their frequent epidemiology in FWS as shown by other groups [19] and (2) the lower likelihood of incidental finding of these viruses in the blood [4]. Second, there was a limited number of SBIs, even though our dataset was sufficient to show the prognostic accuracy of viremia in predicting the absence of SBI. Nevertheless, most of the SBIs were UTIs which can be reliably excluded with a negative urinalysis, and we could not evaluate how viremia performed to exclude other types of SBI given their rarity in our dataset. Third, one cannot formally exclude that some patients with HHV-6 viremia had chromosomally integrated HHV-6, even though this is very unlikely given the low viral load [34]. Then, because of a limited number of patients infected with a given virus, we did not take into account the importance of the viral load to possibly refine the prognostic accuracy. Also, the study was mainly offered to children in whom blood testing was planned, possibly generating an inclusion bias. However, the current study demonstrated the benefit of viral blood tests in children in whom SBI had to be excluded and in whom blood tests were required anyway for clinical purposes. Next, even though the various final diagnoses among non-SBI patients reflects clinical practice in patients with FWS, further perspectives could focus on subgroup analyses based on final diagnosis, which is outside the scope of the current study. Finally, a third of children in our dataset consulted within 12 h of fever onset, which might be associated with falsely negative biomarkers despite an SBI. This could possibly have overestimated the difference in sensitivity between viremia and CRP/PCT, even though early consultation to the PED reflects real-life conditions.

In conclusion, we showed that in FWS, viremia displayed a better independent prognostic accuracy to discriminate children with or without SBI when compared to commonly used biomarkers and could potentially be used in conjunction with CRP and/or PCT in the evaluation of children with FWS. Larger studies should evaluate the role and cost-effectiveness of point-of-care testing of viruses by (RT)-PCR in the plasma or whole blood in management algorithms of children with FWS.

## Supplementary Information

Below is the link to the electronic supplementary material.

Supplementary file1 (XLSX 12 KB)

Supplementary file2 (XLSX 11 KB)

Supplementary file3 (XLSX 10 KB)

Supplementary file4 (XLSX 10 KB)

Supplementary file5 (DOCX 78 KB)

## Data Availability

De-identified individual participant data (including data dictionaries) will be made available, in addition to study protocols, the statistical analysis plan, and the informed consent form. The data will be made available upon publication to researchers who provide a methodologically sound proposal for use in achieving the goals of the approved proposal. Proposals should be submitted to arnaud.lhuillier@hcuge.ch.

## Abbreviations

AdV: Adenovirus
AUC: Area under the curve
CFU: Colony-forming unit
CRP: C-reactive protein
FWS: Fever without a source
HEV: Human enterovirus
HHV-6: Human herpesvirus type 6
HPeV: Human parechovirus
OR: Odds ratio
PED: Pediatric emergency department
PCR: Polymerase chain reaction
PCT: Procalcitonin
POCT: Point of care testing
ROC: Receiver operating characteristic
RT: Reverse transcription
SBI: Serious bacterial infection
UTI: Urinary tract infection
