# Supplementary material for: Viremia as a predictor of absence of serious bacterial infection in children with fever without source
Source: Eur J Pediatr. 2022 Nov 18;182(2):941–7. doi: 10.1007/s00431-022-04690-7 (PMC9672567; doi:10.1007/s00431-022-04690-7)
Supplement: Supplementary file 5 — Supplementary file5 (DOCX 78 KB) [file 431_2022_4690_MOESM5_ESM.docx]

Supplementary Methods. Institutional protocol for fever without source in children 0-3 (A) and 3-36 months (B)

**A**

**FEVER WITHOUT SOURCE**

**0 - 3 MONTHS**

Age <1 month

YES

NO

Systematic

CBC, CRP, PCT

Urinalysis and culture

Blood culture

Lumbar puncture

On an individual basis

Other investigations*

YES

Toxic-appearing child

Lab-score

PCT (ng/ml) Points

< 0,5 0

≥ 0,5 2

≥ 2 4

CRP (mg/l)

< 40 0

40-99 2

≥ 100 4

Urinalysis

negative 0

positive 1

NO

Systematic

CBC, CRP, PCT

Urinalysis and culture

On an individual basis

Other investigations*

Lab-score <3

Lab-score ≥3

Blood culture

Lumbar puncture

No further investigation

**B**

**FEVER WITHOUT SOURCE**

**3-36 MONTHS**

Toxic- appearing child

NO

YES

Systematic

CBC, CRP, PCT

Urinalysis

Case-to-case

Chest X-ray

Systematic

CBC, CRP, PCT

Urinalysis and culture

Blood culture

Lumbar puncture

On an individual basis

Other investigations*

Lab score <3

Lab score ≥3

Systematic

Blood culture

Urine culture

On an individual basis

Other investigations*

Lumbar puncture

No further investigations

Lab-score

PCT (ng/ml) Points

< 0,5 0

≥ 0,5 2

≥ 2 4

CRP (mg/l)

< 40 0

40-99 2

≥ 100 4

Urinalysis

negative 0

positive 1

* Any other additional workup performed on an individual basis (i.e chest X-ray, stool (RT-)PCRs, MRI/CT, nasopharyngeal (RT-)PCRs)

CRP:C-reactive protein, CBC: complete blood count; PCT procalcitonin
